# A rapid review of mental and physical health effects of working at home: how do we optimise health?

**DOI:** 10.1186/s12889-020-09875-z

**Published:** 2020-11-30

**Authors:** Jodi Oakman, Natasha Kinsman, Rwth Stuckey, Melissa Graham, Victoria Weale

**Affiliations:** Centre for Ergonomics and Human Factors; Department of Public Health, La Trobe University, Kingsbury Drive, Bundoora, 3086 Australia

**Keywords:** Working at home, Telecommute, Physical health, Mental health, Gender

## Abstract

**Background:**

The coronavirus (COVID-19) pandemic has resulted in changes to the working arrangements of millions of employees who are now based at home and may continue to work at home, in some capacity, for the foreseeable future. Decisions on how to promote employees’ health whilst working at home (WAH) need to be based on the best available evidence to optimise worker outcomes. The aim of this rapid review was to review the impact of WAH on individual workers’ mental and physical health, and determine any gender difference, to develop recommendations for employers and employees to optimise workers’ health.

**Method:**

A search was undertaken in three databases, PsychInfo, ProQuest, and Web of Science, from 2007 to May 2020. Selection criteria included studies which involved employees who regularly worked at home, and specifically reported on physical or mental health-related outcomes. Two review authors independently screened studies for inclusion, one author extracted data and conducted risk of bias assessments with review by a second author.

**Results:**

Twenty-three papers meet the selection criteria for this review. Ten health outcomes were reported: pain, self-reported health, safety, well-being, stress, depression, fatigue, quality of life, strain and happiness. The impact on health outcomes was strongly influenced by the degree of organisational support available to employees, colleague support, social connectedness (outside of work), and levels of work to family conflict. Overall, women were less likely to experience improved health outcomes when WAH.

**Conclusions:**

This review identified several health outcomes affected by WAH. The health/work relationship is complex and requires consideration of broader system factors to optimise the effects of WAH on workers’ health. It is likely mandated WAH will continue to some degree for the foreseeable future; organisations will need to implement formalised WAH policies that consider work-home boundary management support, role clarity, workload, performance indicators, technical support, facilitation of co-worker networking, and training for managers.

## Background

The current global pandemic caused by coronavirus disease 2019 (COVID-19) has resulted in an unprecedented situation with wide ranging health and economic impacts [1, 2]. The working environment has been significantly changed with thousands of jobs lost and women impacted at higher rates than men [3, 4]. For those employed in sectors able to work remotely, mostly white-collar professional workers, their homes have now become their workplace, school, and place for relaxation. As economies begin to reopen with resumption of some normal activities, questions arise about the potential return to formal office environments and the implications for employees whilst COVID-19 remains active in the community [5]. Many organisations will continue mandating working at home (WAH) for the foreseeable future to avoid making COVID-19 regulation related changes to their office environments [6].

The emergence of new technologies has revolutionised working patterns, enabling work from anywhere for many employees [7, 8]. The concept of telework has existed since the 1970s but in a more limited scope than is currently possible [7]. The extensive availability of technology has enabled location and timing of work to be undertaken with significant flexibility, offering benefits to employers and employees. However, to date there is no universally accepted definition of telework. The International Labour Organisation (ILO) defines telework as the use of information and communications technologies (ICTs) including smartphones, tablets, laptops or desktop computers for work that is performed outside the employer’s premises [7]. A range of positive benefits are associated with teleworking, including improved family and work integration, reductions in fatigue and improved productivity [9]. However, the blurring of physical and organisational boundaries between work and home can also negatively impact an individual’s mental and physical health due to extended hours, lack of or unclear delineation between work and home, and limited support from organisations [10]. The mandatory WAH situation is complex and requires a systematic examination to identify the impact of organisational, physical, environmental and psychosocial factors on individuals’ mental and physical health.

The ongoing need for containment of COVID 19 and continued need to undertake WAH requires evidence synthesis to develop policies and guidelines to protect employees’ health and well-being. We undertook a rapid review of the evidence on the impact of WAH on individual workers’ mental and physical health. In addition, we examined any gender differences of these impacts. We considered the body of evidence to develop recommendations for employers to optimise the health of their employees.

## Methods

### Search strategy and selection criteria

This rapid review was undertaken using principles recommended by the WHO [11]. PRISMA reporting guidelines were followed [12]. The search strategy was developed in consultation with a senior librarian and, for this rapid review, was limited to three databases. ProQuest (Central, Coronavirus Research Database, Social Science Premium Collection, Science Database), PsycINFO and Web of Science databases were searched on 5 May 2020. The search strategy was limited to English language, peer reviewed journal articles published from January 2007 onwards. To ensure wide capture of the literature, study design was not restricted. The date limit was selected to ensure the contemporary work environment was captured. The year 2007 was when the first smartphone was released, this technology change enabled greater flexibility in relation to work arrangements. To ensure the search strategy addressed the research questions two broad concepts were included, those relating to WAH (e.g., “home work”, “telecommute”) and health-related outcomes (e.g., “musculoskeletal risk”, “mental health”). Refer to Appendix for the full search strategy.

For inclusion in the current rapid review, studies were required to focus on adult white collar/professional employees WAH during business hours, and to include mental or physical health related outcomes of workers. Studies were excluded if they focused on domestic workers, self-employed workers, informal working from home (working from home after hours to catch up on work), productivity outcomes, chronic illness/disability, or pregnancy/breast feeding. The rationale of the search strategy was to capture studies which included participants who undertook working from home on a regular basis, but these arrangements did not necessarily have to be mandated or formalised by the organisation.

Titles, abstracts and full texts were screened by two authors using Covidence [13]. Disagreements were resolved by consensus. Reasons for exclusion of studies were noted. The outcomes of interest were measurable changes in physical or mental health. Secondary analysis was undertaken for studies which reported differences by gender.

#### Data extraction and quality assessment

Data extraction was undertaken using a standardised form and included setting, study design, method used, details of participants, industry setting, measures used, and the health outcomes. The risk of bias assessment was used as a proxy for the quality of the study and undertaken for both qualitative and quantitative studies using separate forms. The risk of bias domains were derived from the RTI research bank, Cochrane Collaboration tool quality assessment, and the Johanna Briggs appraisal tool for qualitative research [14–16]. Each potential source of bias was assessed as high, moderate, low, or unclear risk with justification given for judgement. In line with rapid review principles, data extraction and risk of bias for each article was undertaken by at least one author, with a sub sample screened by a second author for accuracy.

An overall quality assessment of each study was determined using a previously published rating system [17]. Studies with a 'low' risk rating for the confounding factors criteria and a higher number of ‘low’ risks than ‘high’ or ‘unclear’ risks, were deemed to have a 'low' overall risk of bias. Studies with a 'high' risk rating for the confounding factors criteria and more ‘low’ risks than ‘high’ or ‘unclear’ risks, were assessed as having 'moderate' overall risk of bias. Studies with a 'high' risk of bias rating for confounding factors criteria, and more ‘high’ or ‘unclear’ risks than ‘low’ risks were designated to have a 'high' overall risk of bias.

### Data analysis

Qualitative data were organised using narrative synthesis to identify how WAH influenced employees physical and mental health. Studies were grouped by broad health outcomes and then a separate analysis by gender undertaken.

## Results

**Fig. 1:**
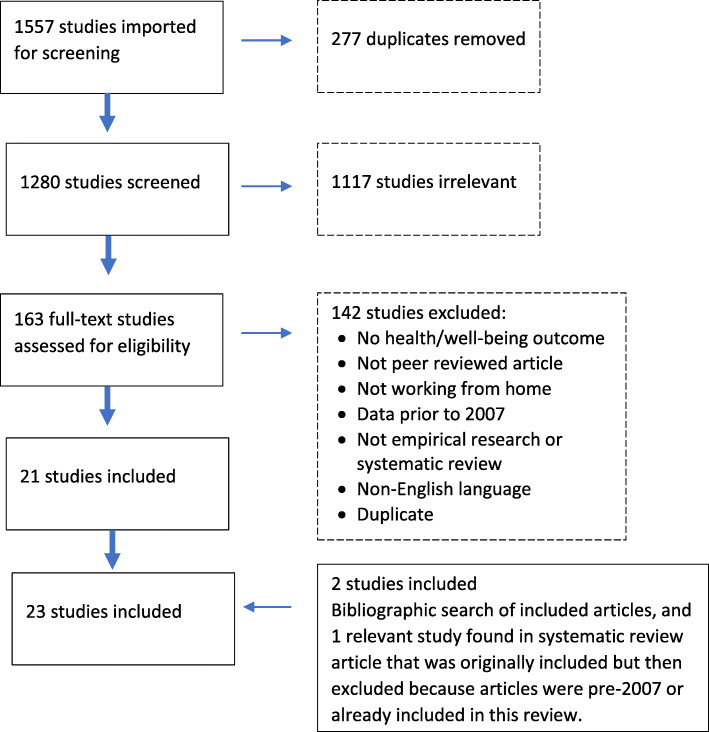
PRISMA diagram

The database search identified 1557 papers of which 21 met the inclusion criteria. Two additional studies were included following a reference list search of the articles which met the inclusion criteria, making a total of 23 studies. The primary reason for exclusion was the study did not include a health outcome. The PRISMA diagram outlines the screening process (see Fig. 1). The studies represented 10 countries (USA, UK, Australia, New Zealand, Japan, Belgium, South Africa, Brazil, Germany, The Netherlands), and varied in study design: 20 cross sectional, one cohort, one controlled before and after, and one combined cross sectional and cohort (refer Table 1). No randomised trials were identified. Studies included 19 quantitative, 3 qualitative and 1 mixed methods.

**Table 1:** Study characteristics

Author (Date) Country of study	Study design (method)	Participants	Measures	Outcome
Anderson et al. (2015) [18] USA	Cross sectional (quant)	102 Federal agency employees (51 F)	Job-Related Affective Well-Being Scale (10 items)	Telework was associated with greater positive effect and lower negative effect on well-being than working in the office. Individual differences were highly variable and related to people’s tendency to ruminate and their social connectedness. Those who were more likely to ruminate experienced more negative affect. People who were more socially connected tended to have even more positive effect and less negative effect on well-being than those who were not as connected.
Bosua et al. (2013) [19] Australia	Cross sectional (qual)	28 employees various sectors (unspecified F)	Interviews and 4-day diary entry (unvalidated measures)	Employees working at home some of the time reported greater sense of well-being and less stress on days they were working at home; preferred hybrid teleworking so could have social interaction and networking at office.
Bentley et al. (2016) [20] New Zealand	Cross sectional (quant)	804 teleworkers various sectors (378 F)	Psychological strain (GHQ-12) Teleworker support (14 items covering manager support, trust and technical support)	Increased organisational and manager support for teleworkers reduces psychological strain.
Eddleston & Mulki (2017) [21] USA	Cross sectional (mixed methods)	Qual: 52 employees various sectors (32 F) Quan: 299 employees technology company (132 F)	Semi structured interviews on experiences and challenges of remote workers. Questionnaire including: Job stress, Work-family integration, Inability to disengage from work	Working from home associated with inability to disengage from work and integration of work into the family domain. Inability to disengage from work is positively related to job stress, via increased WFC. This effect is greater for women than men. Integration of work into the family is positively related to job stress via increased WFC. This effect is greater for men than women.
Filardí et al. (2020) [22] Brazil	Cross sectional (quant)	98 teleworkers public administration (40 F)	Questionnaire (1 item quality of life, 1 item safety)	95% participants reported improved quality of life, perceived increased safety when working from home and reduced stress associated with commuting
Gimenez-Nadal et al. (2020) [23] USA	Cross sectional (quant)	2471 employees from various sectors. (1106 F)	American Time Use Survey 2012–2013 (diaries and 5 item well-being questionnaire)	Male teleworkers reported statistically significant lower levels of stress, pain & tiredness compared to commuters. Female teleworkers reported higher levels of happiness compared to commuters but for stress, pain and tiredness no statistically significant differences were found.
Golden (2012) [24] USA	Cross sectional (quant)	316 employees of a large computer company (92 F)	Work exhaustion (5 items from General Burnout Questionnaire)	Those who teleworked extensively, when WFC was high, were more exhausted compared to those who did limited telework. When WFC was low, they had lower exhaustion than those who did limited telework.
Grant et al. (2013) [25] UK	Cross sectional (qual)	11 employees various sectors (7 F)	Semi-structured interview covered e-working and well-being	Communication and support from colleagues emerged as two key factors to ensure successful remote working and to balance the psychological aspects of well-being. Sub-themes of building relationships and interacting, including where boundaries could be crossed over, were considered to positively affect psychological well-being.
Hayman (2010) [26] New Zealand	Cross sectional (quant)	336 administration university employees	Validated job induced stress measure (7 items)	Working from home was associated with lower job induced stress.
Henke et al. (2016) [8] USA	Cross sectional (quant)	3703 financial employees (2296 F)	Single item stress and depression measure	Lower hours of telecommuting associated with lower levels of depression. No time dependent relationships of telecommuting with stress.
Hornung & Glaser (2009) [27] Germany	Cross sectional (quant)	1008 public administration employees (277 F)	World Health Organisation-BREF Quality of Life survey (6 items)	Increased telecommuting improved quality of life through increased autonomy (mediator). Analysis by gender found the relationship remained for males but not for females.
Kaduk et al. (2019) [28] USA	Cross sectional (quant)	Fortune 500 organisation, IT workforce 758 non-supervisory employees	Validated scales: Emotional exhaustion (3 items),	Voluntary remote work is protective with regard to stress. Involuntary remote work associated with higher emotional exhaustion than those undertaking voluntary remote work.
Kazekami (2020) [29] Japan	Cohort (quant)	9200 employees various sectors (61% regular teleworkers)	Measure of stress and happiness (single item)	Telework was associated with increased stress, and increased happiness, for males, no effect for females.
Kim et al. (2020) [30] USA	Cross sectional (quant)	Previously collected data, US Quality of Work survey, 6945, (3599 F)	General Social Survey & Quality of Work Life survey data (Job stress measure single item, Daily fatigue measure single item)	Working at home associated with decreased fatigue and stress compared to those not working at home. No significant gender differences.
Major et al. (2008) [31] USA	Cross sectional (mixed)	863 Federal government employees with dependents, (630 F)	19 item web-based survey (designed by researchers, unvalidated)	89% telecommuters were less stressed and 77% had increased energy levels compared to when they worked in the office.
Nijp et al. (2016) [32] The Netherlands	Case control (quant)	1443 Financial company employees (teleworkers vs non-teleworkers) (521 F)	Fatigue Assessment Scale (3 items), Health question (10-point scale, 1 item)	After intervention (working from home), small decrease in self-reported health but no effect on fatigue.
Sardeshmukh et al. (2012) [33] USA	Cross Sectional (quant)	417 supply chain management company employees (121 F)	Survey including validated exhaustion measure (8 item)	Telework negatively related to exhaustion, directly and indirectly via job demands and resources.
Song & Gao (2019) [34] USA	Cross sectional (quant)	3962 Full time workers who participated in the 2010, 2012 and 2013 American Time Use Survey Well-Being Modules. (1277 F)	American Time Use Survey Subjective Well-Being (SWB) Scale (6 items)	Telework associated with increased stress and decreased happiness regardless of when it occurred. Teleworking on weekdays, fathers reported higher level of stress, pain and reduced happiness. For mothers, teleworking results in reduced happiness and increased fatigue.
Suh & Lee (2017) [35] South Korea	Cross sectional (quant)	256 IT company teleworkers (111 F)	Survey using various adapted validated scales. 31 items	Teleworkers working less than 2.5 days per week experienced greater strain from technostress (work overload, role ambiguity & invasion of privacy) compared to those working greater than 2.5 days per week.
Tietze & Nadin (2011) [36] UK	Cohort (qual)	7 employees local tax department (7F)	3 guided interviews with each participant, analysed using ‘template analyses’	Employees WAH reported enhanced personal well-being compared to working in the office.
Tustin (2014) [37] South Africa	Cross sectional (quant)	Academic telecommuters (*n* = 36) Academic non-telecommuters (*n* = 102)	46 web-based survey items (researcher constructed)	Telecommuters reported improved quality of life, healthier eating, and reduced work-related stress. Teleworkers had less emotional and physical fatigue than non-teleworkers.
Vander Elst (2017) [38] Belgium	Cross sectional (quant)	828 Telecommunication company employees (99 F)	Utrecht Burnout Scale (emotional exhaustion 5 items) COPSOQ (cognitive stress scale, 5 items)	Extent of telecommuting did not directly relate to emotional exhaustion or cognitive stress. More days of telecommuting associated with higher levels of emotional exhaustion and cognitive stress due to less social support from colleagues.
Windeler et al. (2017) [39] USA	Study 1 Cohort (4 months), Study 2 cross sectional (quant)	Study 1: 51 Financial services firm, IT workers. (20 F) Study 2: 258 employees various sectors (103 F)	Work exhaustion – 4-item scale from the Maslach Burnout Inventory—General Survey	Telework increased the negative effect of external interaction on work exhaustion because communication with external stakeholders requires more effort. Males experienced higher levels of work exhaustion after starting telework compared to those who did not telework (Study 1). Females who teleworked experienced higher levels of work exhaustion compared to those who did not telework (Study 2).

*quant* Quantitative, *qual* Qualitative, *mixed* Quantitative and qualitative, *F* Female

Studies were conducted in the following industry sectors: government departments and agencies (five), financial services (three), technology (two), academia (one), telecommunications (one), logistics (one). Ten studies used data from surveys of the general public or did not focus on a particular industry sector. The number of hours and nature of WAH arrangements varied between studies; participants WAH either full time (two studies [21, 36] or part-time, and had access to a formal WAH policy or ad hoc WAH approval by managers. Only one study examined employees undertaking mandatory WAH [36]. Some studies did not specify the nature of the WAH arrangements. Due to the heterogenous nature of the studies, it was not possible to conduct a meta-analysis.

### Health related outcomes

Physical health related outcomes (*n* = 3) identified in the studies included: pain, self-reported health and perceived safety. Mental health related outcomes (*n* = 7) identified included: well-being, stress, depression, fatigue, quality of life, strain and happiness. Seven studies undertook separate gender analysis (see Table 2).

**Table 2:** Summary of studies by health outcome

	Pain	Self-reported health	Safety	Well-being	Stress	Depression	Fatigue	Quality of life	Strain	Happiness	Gender analysis
Anderson (2015) [18]				✓							
Bosua (2013) [19]				✓	✓						
Bentley (2016) [20]									✓		
Eddleston & Mulki (2017) [21]					✓						✓
Filardí (2020) [22]			✓		✓			✓			
Gimenez-Nadal (2020) [23]	✓				✓		✓			✓	✓
Golden (2012) [24]							✓				
Grant (2013) [25]				✓							
Hayman (2010) [26]					✓						
Henke (2016) [8]					✓	✓					
Hornung & Glaser (2009) [27]								✓			✓
Kaduk (2019) [28]					✓		✓				
Kazekami (2020) [29]					✓					✓	✓
Kim (2020) [30]					✓		✓				✓
Major et al. (2008) [31]					✓		✓				
Nijp (2016) [32]		✓					✓				
Sardeshmukh (2012) [33]							✓				
Song & Gao (2019) [34]	✓				✓		✓			✓	✓
Suh & Lee (2017) [35]									✓		
Tietze & Nadin (2011) [36]				✓							
Tustin (2014) [37]					✓		✓	✓			
Vander Elst (2017) [38]				✓	✓						
Windeler (2017) [39]							✓				✓

### Risk of bias

Following assessment of risk of bias, quantitative studies were rated as: four high risk, three moderate risk, and 13 low risk. For the qualitative studies (*n* = 3) the overall risk of bias for all studies was assessed as moderate. The four studies with high risk of bias included cross sectional surveys [18, 22, 26, 31]. For the cohort studies, quantitative [29], qualitative [36] and mixed methods [39] were utilised, with moderate and low risk of bias, respectively (see Tables 3 and 4).

**Table 3:** Quality assessment of quantitative studies

	Inclusion criteria	Recruitment	Ethics	Protocol	Blinded assessors	Measures	Follow up	Outcome data	Selective reporting	Confounders	Other	Overall Risk
Anderson et al. (2015) [18]	–	N/A	?	N/A	N/A	?	N/A	+	–	?	?	High
Bentley (2016) [20]	–	N/A	+	–	N/A	–	N/A	+	–	?	–	Mod
Eddleston (2017) [21]	–	–	–	N/A	N/A	–	N/A	–	–	–	–	Low
Filardi (2020) [22]	–	N/A	?	N/A	N/A	+	N/A	?	?	+	+	High
Hayman (2010) [26]	?	N/A	?	N/A	N/A	–	N/A	?	–	+	+	High
Henke (2016) [8]	–	–	?	–	–	+	N/A	–	–	–	–	Low
Hornung (2009) [27]	–	N/A	?	+	N/A	–	N/A	?	–	–	–	Low
Gimenez (2020) [23]	–	N/A	–	+	N/A	–	N/A	–	–	–	+	Low
Golden (2012) [24]	–	N/A	?	N/A	N/A	–	N/A	–	–	–	–	Low
Kaduk (2019) [28]	–	N/A	?	N/A	N/A	–	N/A	+	–	–	–	Low
Kazekami (2020) [29]	?	?	?	N/A	N/A	?	N/A	?	–	–	–	Mod
Kim (2019)	–	N/A	+	–	N/A	–	N/A	–	–	–	+	Low
Major (2008) [31]	–	N/A	?	–	N/A	+	N/A	?	–	+	–	High
Nijp (2016) [32]	–	–	+	–	+	–	–	–	–	–	–	Low
Sardeshmukh (2012) [33]	–	N/A	+	N/A	N/A	–	N/A	–	–	–	–	Low
Song (2019) [34]	–	–	–	N/A	N/A	–	N/A	–	–	–	–	Low
Suh (2017) [35]	–	N/A	+	–	N/A	–	N/A	–	–	–	–	Low
Tustin (2014) [37]	–	–	?	–	N/A	–	N/A	–	–	+	–	Mod
Vander Elst (2017) [38]	+	–	–	N/A	N/A	–	N/A	?	–	–	–	Low
Windeler (2017) [39]	–	N/A	+	N/A	N/A	–	N/A	?	–	–	–	Low

(−) = low risk; (?) = unclear (if the study did not provide adequate details of the criteria in question, the rating was deemed unclear); (+) = high risk; *N/A* Not applicable

**Table 4:** Quality assessment of qualitative studies

	Inclusion criteria	Method & aim congruity	Method & data collection congruity	Participant voices	Ethics	Protocol	Measures	Outcome data	Outcome reporting	Confounders	Other	Overall
Bosua (2013) [19]	–	–	–	–	+	–	+	–	+	+	+	Mod
Grant (2013) [25]	–	–	–	–	+	+	+	–	–	+	+	Mod
Tietze (2011) [36]	–	–	–	–	+	–	–	+	–	+	+	Mod

(−) = low risk; (+) = high risk

### Physical health-related impacts

Three studies explored the physical health impacts of WAH [22, 23, 32]; one of these will be discussed in the section on gender. Filardí [22] surveyed government employees who reported that ‘I feel safer working from home’, but the WAH arrangements were not clearly defined. In contrast, a study by Nijp et al. [32] found WAH had a negative impact on physical health. This study measured self-reported health in a control and an intervention group of finance company employees, before and after implementation of a policy to enable part-time WAH. Participants reported a small but statistically significant decrease in self-reported health which could not be explained as usual health indicators and job demands remained unchanged.

### Mental health-related impacts

The majority of studies (21 studies) explored the effect of working at home on mental health. Fourteen are explored in this section and seven studies that included a gender analysis are presented separately.

The impacts of WAH on mental health were complex. Nine studies considered environmental, organisational, physical, or psychosocial factors in the relationship between WAH and mental health [18, 20, 21, 24, 25, 31, 33, 35, 38]. Working at home could have negative or positive impacts, depending on various systemic moderators such as: the demands of the home environment, level of organisational support, and social connections external to work.

Five studies [20, 25, 33, 35, 38] examined the influence of colleagues and organisational support on WAH. Suh & Less [35] compared the effect of technostress (defined as work overload, invasion of privacy, and role ambiguity) on IT company employees doing low intensity WAH (< 2.5 days per week), to those doing high intensity WAH (> 2.5 days per week). Low intensity WAH employees experienced higher strain associated with work overload and invasion of privacy, related to IT complexity, pace of IT change, lower job autonomy, and being constantly in electronic contact with work. Bentley et al. [20] explored the influence of organisational (social and manager) support on health outcomes of WAH employees and found a similar relationship between lower levels of organisational support and higher psychological strain. Sardeshmukh et al. [33] also examined the effects of organisational support (via job resources and demands) and found associations between WAH and less time pressure, less role conflict, and greater autonomy, resulting in less exhaustion. However, they also found WAH was associated with lower social support, lower feedback and greater role ambiguity which increased exhaustion; overall these negative effects did not outweigh the overall positive impact of WAH. Vander Elst et al. [38] found increased WAH hours were associated with less emotional exhaustion and cognitive stress which was mediated by support from colleagues. Those working more days at home experienced greater emotional exhaustion and cognitive stress associated with reduced social support from their colleagues. Grant et al. [25] interviewed employees WAH and identified colleagues’ support and communication as important influences on psychological well-being. Tietze et al. [36] interviewed seven employees WAH on a full-time basis as part of a three-month pilot scheme. Employees reported an improved sense of personal well-being as they were no longer in a stressful office environment.

Anderson [18] measured the effect of WAH on the mental well-being of government employees (all participants were WAH > 1 day per fortnight), and found WAH had a positive effect on well-being (feeling at ease, grateful, enthusiastic, happy, and proud) with less negative effect on well-being (bored, frustrated, angry, anxious, and fatigued). The study also found individual traits of openness to experience, lower rumination, and greater social connectedness moderated the relationship between WAH and positive well-being, and a strong level of social connectedness (outside of work) was related to a less negative effect on well-being.

Two studies explored the home environment as a mediator for the relationship between WAH and health related outcomes. Work-family conflict (WFC) occurs when the demands of work impinge on domestic and family commitments. Golden’s [24] study of computer company employees who were WAH for greater periods of time than in the office, found high levels of exhaustion when combined with a high level of WFC. When WFC was low the same employees experienced a low level of exhaustion compared to those WAH only occasionally. Another study [31], which surveyed employees with dependent-care responsibilities, found an association between WAH and increased energy levels, and decreased stress; WAH acted as a mediator between health-related outcomes and dependent care responsibilities.

Relationships between WAH and the following mental health-related outcomes were examined: stress [8, 19, 21–23, 26, 28–31, 34, 37, 38], quality of life [22, 27, 37], well-being [18, 19, 25, 36, 38], and depression [8]. Five studies [19, 26, 28, 31, 37], reported a decrease in stress levels of employees WAH on a part-time basis. One study [8] explored employees who were WAH either all or part of their work time and found no direct relationship between WAH and levels of stress. In contrast, VanderElst et al. [38] found WAH was associated with increased stress. Quality of life was enhanced through WAH in two surveys of employees [22, 37]. Filardí et al. [22] included public sector employees but did not report how long employees were WAH. Tustin [37] included university employees who were WAH for some of the week.

Bosua et al. [19] studied employees from government, education and private sectors WAH for some of their week and found a greater sense of well-being was reported compared to when working in the office. Of note, participants reported their preference was to combine WAH with some office time so they could connect with colleagues.

Henke et al. [8] conducted a study within a financial company and compared employees WAH to those not WAH; those WAH less than 8 h per month had statistically lower levels of depression than those not WAH. No statistically significant relationships were identified between depression and greater number of hours WAH.

Four studies examined the direct relationship impact of WAH on fatigue (including exhaustion, tiredness or changes in energy levels) with mixed results [28, 31, 32, 37]. Two studies [31, 37] concluded WAH resulted in decreased levels of fatigue. However, others [28, 32] concluded WAH had no effect on levels of fatigue.

### The gender differences in health outcomes related to WAH

Seven studies examined outcomes by gender [21, 23, 27, 29, 30, 34, 39]. Three studies considered complex interactions when examining gender differences in the WAH and health related outcome relationship. Windelar et al. [39] examined the effect of interpersonal and external interactions on work exhaustion, using WAH as a moderator. They surveyed employees pre and post implementation of a formal WAH policy (study 1) and then compared employees WAH to those based in the office (study 2). Males had higher levels of work exhaustion following the commencement of telework (study 1). Both studies found WAH increased the negative effect of interactions external to the business on work exhaustion. Females WAH reported higher levels of work exhaustion compared to their colleagues who remained at the office (Study 2). Hornung et al. [27] examined the role of mediators on the relationship between WAH and mental health and gender differences; they surveyed public servants and found increased time WAH improved quality of life through increased autonomy (mediator). However, in a separate gender analysis the relationship was only significant for males. Eddleston & Mulki [21] reported an increase in job stress for employees WAH full-time. This was mediated by WFC; an inability to disengage from work, and the integration of work into home life, led to higher WFC which was associated with higher job stress. This relationship was moderated by gender with women experiencing greater WFC due to inability to disengage from work, and men experiencing greater WFC due to integration of work into the family domain.

The remaining four studies examined the direct relationship between WAH and health outcomes. Two studies, both using data from the American Time Use Survey, examined physical and mental health outcomes by gender. Gimenez-Nadal et al. [23] identified participants WAH as those who indicated non-commute days in a diary record. Diary records were followed by a well-being survey, where male teleworkers reported lower pain levels, lower stress, and lower tiredness *(p* < 0.05) compared to non-teleworkers; no differences were found between female teleworkers and non-teleworkers. Song & Gao [34] compared subjective pain when WAH to work at the office, by gender and parental status, and reported no differences. However, fathers who were WAH reported increased stress, and mothers WAH had decreased happiness.

Kim et al. [30] and Kazekami [29] examined the direct relationship between fatigue, stress and happiness. Kim et al. [30] reported males who were WAH regularly had lower levels of fatigue and stress compared to those who did not. For women, WAH was associated with lower stress levels but higher levels of fatigue compared to those not WAH. Kazekami [29] found that males WAH reported increased stress and happiness whilst no effect was found for females.

## Discussion

Due to the current COVID-19 situation, WAH has been implemented as part of a broad public health measure to prevent the spread of an infectious disease. Although this measure was introduced rapidly, it is likely WAH will remain in place for some time and organisations will utilise this as a strategy to manage the necessary physical distancing requirements to prevent further outbreaks of COVID-19. This rapid review explored the impact of WAH on physical and mental health outcomes to inform the development of guidelines to support employers in creating optimal working conditions. In addition, included studies were examined to explore any gender differences in the relationship between WAH and health.

The majority of studies in the rapid review employed cross-sectional designs and were of variable quality. The definition of WAH and the number of days per week employees were working at home were often unclear. Of the 23 studies identified as relevant to this review, only one investigated the condition of mandatory WAH [36], the remainder involved workers who were electing to WAH for different but regular time periods across a week. However, evidence from the review does suggest there are some reasonable actions employers can take to support their employees in optimising their working conditions whilst at home. This discussion will outline the physical and mental health outcomes of WAH and then, drawing on these findings, outline implications for practice.

Physical health and WAH was only examined in three studies. The very low number of studies identified could suggest the search strategy was not adequately targeted to capture studies assessing the physical health outcomes of WAH; however, a range of terms associated with musculoskeletal health were included. Grey literature may have offered further insights but was not included in this rapid review. An alternative explanation may be that in cases where employees are working at home for limited time periods, the use of standard guidelines for workstation arrangements have been considered sufficient and deployed to manage the physical health of workers. The limited coverage of physical health outcomes of WAH was not expected. Previous research, in relation to the occupational health of employees, suggests the focus is more typically on the physical aspects of health [40].

In contrast, the impact of WAH on mental health outcomes was covered by the majority of included studies (*n* = 21). Three of the studies employed longitudinal approaches [29, 36, 39] with mixed results such as increased stress [29], improved well-being [36] and gendered impacts on exhaustion levels [39]. The mixed results and varying quality of the articles does create challenges in drawing out meaningful themes; however, differences in organisational responses and support were identified as important contributors to either increasing or mitigating negative health outcomes (e.g. [20, 25, 33, 35]). The complexity of the WAH situation received only limited coverage [20]. An extensive literature exists supporting the important role of the work environment (e.g. leadership, collegial support, job design) on employees’ health [41]. The translation of this body of work undertaken in conventional office environments has not yet been undertaken in WAH and offers opportunities for further research.

Only one third of the studies (*n* = 7) undertook separate analysis by gender on the impacts of WAH on health. The differences in health impacts may reflect traditional gender roles where males are perceived as ideal citizen workers whose primary focus is work, whilst for women dual roles exist in the work and domestic sphere which remains pervasive in many cultures [42]. The situation of WAH may challenge the ability to separate these roles, creating conflict due to the lack of physical distance enabled by undertaking work outside of the house. High levels of WFC are associated with negative outcomes, including poor mental and physical health [43, 44], and it is plausible that for some females this is exacerbated by the WAH situation, contributing to the higher levels of exhaustion and stress reported by females [21, 39] compared to males in WAH and, for some, increased unhappiness [34].

### Implications for practice

Drawing on the evidence from the current rapid review, key themes were identified and are provided here as considerations to assist with developing optimal working conditions for employees WAH, including organisational support, co-worker support, technical support, boundary management support, and addressing gender inequities:

#### Organisational support

The current pandemic situation has resulted in many sudden and unexpected changes to work practices which potentially create uncertainty for employees, necessitating regular communication to ensure clarity around role expectations, clearly defined performance measures, appropriate workloads, and access to human resources support [19, 24, 26, 28, 33]. Systems which optimise regular, reliable, and consistent communication, using methods which are appropriate for employers and employees, need to be negotiated and implemented. In addition, organisations need to provide training and assistance for managers supervising WAH employees [22, 25, 31]. Organisations may also consider financial compensation to employees for costs associated with WAH [31].

#### Coworker support

WAH can be isolating with employees feeling disconnected from their managers and colleagues. Systems which facilitate effective formal and informal coworker support are needed. Formal coworker support that occurs in teams when people are collocated, such as sharing of tasks and incidental problem solving, requires facilitation whilst WAH. In the current mandated WAH situation, provision of regular face-face online contact opportunities and social support could replace the day in the office [20, 24, 32, 38]. In situations where WAH becomes voluntary, employees are likely to benefit from a regular day in the office to maintain networks [19, 32, 38].

#### Technical support

The sudden and unexpected requirement to undertake technologically dependent work roles within the domestic environment has exposed the need for high quality technology services for those WAH. Effective WAH requires the provision of appropriate equipment and high-quality technology support in conjunction with training in the necessary software and systems needed by an individual [19, 22, 31, 37].

#### Boundary management support

Although only one study reported on mandated WAH [28], other studies investigated the impact on boundaries between work, domestic, and recreational boundaries [21, 24, 35]. To facilitate boundary management, clarity is required in relation to the expectations of working hours to prevent employees feeling as though they are ‘on call 24/7’ [30]. Strategies to facilitate this could include education of employees and managers on how to more formally develop boundaries between work and family [21].

#### Addressing gender inequities

A key policy priority to support WAH should be targeted at the development of adaptable strategies to ensure they meet the nuanced needs of different employees, irrespective of gender or life course stage. Strategies also need to ensure those who choose or are mandated to work at home do not experience negative career consequences, such as not being offered career advancement or training opportunities [45, 46].

### Study limitations

Strengths and limitations of this review must be considered. Despite this being a rapid review, a systematic procedure for searching and selection of articles was retained. A further strength was the undertaking of a formal quality appraisal along with reference checks of included studies to reduce the likelihood of omitting relevant literature. However, the review was limited to English language peer-reviewed publications and no search of the grey literature was undertaken. We excluded studies which did not contain a health outcome as a separate measure, therefore some studies which were in the domain of working at home but examined other outcomes, such as productivity, were excluded. Only one study on mandatory WAH was identified, hence the inclusion of studies which examined the voluntary situation were retained. Heterogeneity of outcome measures across studies make direct comparisons difficult; as such a meta-analysis was not undertaken. Retention of all study types and methodologies was undertaken to fully capture data on WAH. Due to the time constraints of this review, contact of authors for additional information was not possible. This review has made several recommendations to support employees WAH, based on the reviewed literature; however, caution is warranted in relation to the unknown impact of the mandatory WAH, which is a unique situation. In the interim , consolidation of the available literature is required, along with longitudinal research to identify causal relationships between WAH and health outcomes.

## Conclusion

Overall, the findings from this review suggest the impacts of WAH on individuals’ mental and physical health vary considerably. However, despite limitations with a relatively low number of studies, some consistent principles emerge which can be used to support employers in improving working conditions to mitigate the negative effects of WAH, and enhance the positive effects of WAH on employees’ health. At a minimum, opportunity for regular communication between managers and their team and between colleagues are important and help to reduce the negative impacts associated with feeling isolated whilst WAH. In situations where WAH continues to be mandatory, consideration of the impact on the home environment and the financial impacts of being at home on a full-time basis (e.g., increased heating, cooling and telecommunication costs) is required. Some financial compensation may be appropriate for employees to reduce this fiscal burden, although some of these costs may be offset by reduced costs associated with commuting.

Longitudinal research is required, which systematically considers all factors in the relationship between employees and their organisations whilst WAH; this can inform the development of guidelines to facilitate the creation of optimal WAH conditions to reduce any negative impacts of employees’ health and well-being.

## Data Availability

Not applicable.

## Appendix

**Table 5:** Full search strategy - ProQuest (Central, Coronavirus Research Database, Social Science Premium Collection, Science Database)

Search ID#	Search Terms	Search Notes	Results
S1	noft(“home work*” OR “remote work*” OR telecommut* OR telework* OR “work* from home”) NOT noft(telemetry OR teleoperation OR telemental OR “domestic worker*” OR “homecare worker” OR “medical resident*” OR “residential care” OR “aged care” OR “residential facility” OR “elder care” OR “home care” OR nurs*)		92,419
S2	MAINSUBJECT.EXACT(“Musculoskeletal diseases”) OR MAINSUBJECT.EXACT(“Mental health”) OR noft(“musculoskeletal risk*” OR “musculoskeletal disease” OR “neck pain” OR “neck injury” OR “back pain” OR “back injury” OR “shoulder pain” OR “shoulder injury” OR “wrist pain” OR “wrist injury” OR “arm pain” OR “arm injury” OR “carpal tunnel syndrome” OR “work related injur*” OR “work related musculoskeletal disorder*” OR WMSD* OR MSD* OR “occupational overuse” OR “neck tension” OR “muscular function*” OR “muscle pain” OR “limb disorder*” OR “psychological issue*” OR depression OR anxiety OR stress OR mental health OR “well being” OR happiness OR comfort OR security OR safety OR “good physical condition” OR fitness OR healthiness OR “physical fitness”)		34,075,172
S3	1 AND 2		10,508
S4	Filter - English		10,469
S5	2007–2020		6011
S6	Peer reviewed		327

## Abbreviations

WAH: Working at home
WFC: Work to family conflict
